# Circulating histones are major mediators of systemic inflammation and cellular injury in patients with acute liver failure

**DOI:** 10.1038/cddis.2016.303

**Published:** 2016-09-29

**Authors:** Zongmei Wen, Zhen Lei, Lu Yao, Ping Jiang, Tao Gu, Feng Ren, Yan Liu, Chunyan Gou, Xiuhui Li, Tao Wen

**Affiliations:** 1 Department of Anesthesiology, Shanghai Pulmonary Hospital, Tongji University School of Medicine, Shanghai 200433, China; 2 Medical Research Center, Beijing Chao-Yang hospital, Capital Medical University, Beijing 100020, China; 3 Department of Forth Cadre, Chinese PLA Army General Hospital, Beijing 100700, China; 4 Department of Oncology, First Hospital of Qinhuangdao, Qinhuangdao 066000, China; 5 Beijing Youan Hospital, Capital Medical University, Beijing 100069, China

## Abstract

Acute liver failure (ALF) is a life-threatening systemic disorder. Here we investigated the impact of circulating histones, recently identified inflammatory mediators, on systemic inflammation and liver injury in murine models and patients with ALF. We analyzed histone levels in blood samples from 62 patients with ALF, 60 patients with chronic liver disease, and 30 healthy volunteers. We incubated patients' sera with human L02 hepatocytes and monocytic U937 cells to assess cellular damage and cytokine production. d-galactosamine plus lipopolysaccharide (GalN/LPS), concanavalin A (ConA), and acetaminophen (APAP) were given to C57BL/6N mice to induce liver injury, respectively, and the pathogenic role of circulating histones was studied. Besides, the protective effect of nonanticoagulant heparin, which can bind histones, was evaluated with *in vivo* and *ex vivo* investigations. We observed that circulating histones were significantly increased in patients with ALF, and correlated with disease severity and mortality. Significant systemic inflammation was also pronounced in ALF patients, which were associated with histone levels. ALF patients' sera induced significant L02 cell death and stimulated U937 cells to produce cytokines, which were abrogated by nonanticoagulant heparin. Furthermore, circulating histones were all released remarkably in GalN/LPS, ConA, and APAP-treated mice, and associated with high levels of inflammatory cytokines. Heparin reduced systemic inflammation and liver damage in mice, suggesting that it could interfere with histone-associated liver injury. Collectively, these findings demonstrate that circulating histones are critical mediators of systemic inflammation and cellular damage in ALF, which may be potentially translatable for clinical use.

Acute liver failure (ALF) is a complex condition wherein rapid-onset liver insult results in coagulopathy, hepatic encephalopathy (HE), and even multiple organ failure and death in a patient without previously recognized liver disease.^1, 2^ The causes of ALF vary greatly by geographical region. In the Western countries, acetaminophen (APAP) constitutes nearly 60–70% of the cases, whereas the primary cause of ALF in China is viral hepatitis B, which accounts for 70–80% of the cases.^3, 4^ Despite recent therapeutic advances, ALF remains a serious clinical condition associated with a high mortality rate. The underlying mechanisms of ALF are multifactorial and incompletely understood. Emerging evidence suggests that, regardless of various etiologies of ALF, its acute onset is generally associated with significant and uncontrolled activation of systemic inflammation, which may consequently lead to multiple organ dysfunction and a poorer prognosis in ALF.^5, 6^ It has been accepted that systemic inflammation may have a crucial role in the initiation and progression of ALF, but the key factors or mechanisms that are responsible for its activation in ALF are largely unclear. Therefore, identification of key mediators that may modulate ALF-associated inflammation is highly desirable.

Extracellular histones in the circulation are recently identified as the pivotal mediators in systemic inflammatory diseases.^7, 8, 9, 10^ It reveals that circulating histones have numerous toxic effects including direct cytotoxicity, induction of vascular permeability, coagulation activation, platelet aggregation, and cytokine production,^11, 12, 13^ all of which are possible mechanisms related to the development of inflammatory organ injuries. Furthermore, histone-targeted therapy has promising potentials for the treatment of various inflammatory injuries. We, therefore, investigated circulating histone levels in patients with ALF, with a hypothesis that histones present in the circulation of ALF patients could serve as inflammatory mediators mediating cellular damage and interfering with disease progression and mortality. We further examined whether histone-mediated toxicity could be neutralized by nonanticoagulant heparin, which can bind histones,^14^ through *in vivo* and *ex vivo* translational studies, with an aim to providing an interventional strategy for ALF in clinical practice.

## Results

### Demographic and clinical features

The baseline demographical, laboratory, and clinical data of the patients and the control subjects are listed in Table 1. The causes for ALF in this study included the following: viral factors (HBV, HBV+HDV, and HEV), drug-induced liver injury (APAP, herbal medicine, and idiosyncratic drug), autoimmune hepatitis, and indeterminate (cases in which a specific cause could not be determined). The causes for CLD included the following: chronic hepatitis B, chronic hepatitis C, and liver cirrhosis. On admission, 37 ALF patients had HE grade I or II, and 25 patients had grade III or IV. Of 62 ALF patients, 28 patients survived at twenty-eighth day and 34 patients died. ALF patients had significantly higher admission international normalized ratio (INRs) and serum ALT, total bilirubin (Tbil), creatinine levels, as compared with CLD patients or healthy controls (Table 1).

### Circulating histone levels in ALF patients correlate with disease severity and mortality

**Figure 1:**
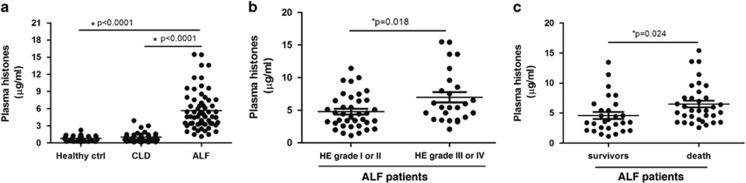
Circulating histones were increased in ALF patients and correlate with disease severity and mortality. (**a**) Median admission plasma histones were significantly higher in ALF patients than in CLD patients or healthy controls (both *P*<0.0001). There was a slight elevation of histone levels in CLD patients in contrast to healthy controls, but the difference was not significant (*P*=0.067). (**b**) ALF patients having HE grade III or IV had higher histone levels than patients having HE grade I or II (*P*=0.018). (**c**) Circulating histone levels were higher in ALF patients who died within 28-day stay than those in survivors (*P*=0.024). Variables were expressed as median (interquartile range)

Median admission plasma histone levels were significantly higher in ALF patients (5.587 *μ*g/ml (0.694, 7.892)) than in CLD patients (1.035 *μ*g/ml (0.578, 1.157)), or healthy controls (0.794 *μ*g/ml (0.378, 1.136)), respectively. Approximately, there was a five- or six-fold increase of histone levels in ALF patients compared with patients with CLD or healthy controls (Figure 1a). There was a slight elevation of histone levels in CLD patients in contrast to healthy controls, but the difference was not significant (*P*=0.067).

Our further analysis showed a clear correlation between histone levels and a number of markers of disease severity or mortality in ALF patients. For example, admission histone levels were associated with ALT levels, INRs, sequential organ failure response syndrome (SOFA), and model for end-stage liver disease (MELD) scores in ALF patients, but weakly correlated with systemic inflammatory response syndrome (SIRS) score, serum Tbil, and serum creatinine levels (Supplementary Table 1). There was a significant difference in admission histone levels in ALF patients having HE grade III or IV compared with the patients having HE grade I or II (5.445 *μ*g/ml (3.927, 8.547) *versus* 4.435 *μ*g/ml (3.015, 6.475), *P*=0.018) (Figure 1b). Moreover, patients with elevated histone levels at the time of admission had increased mortality. There was a significant difference between histone levels in ALF patients who died within 28-day stay compared with survivors (5.415 *μ*g/ml (4.054, 7.561) *versus* 3.531 *μ*g/ml (2.387, 5.703), *P*=0.024) (Figure 1c). In addition, we compared admission plasma histone levels among ALF patients with different etiologies and found no significant difference (all *P*>0.05, data not shown), thus suggesting that the sources of histones in the circulation are not etiologies specific.

### Correlation of circulating histones with systemic inflammation in ALF patients

**Figure 2:**
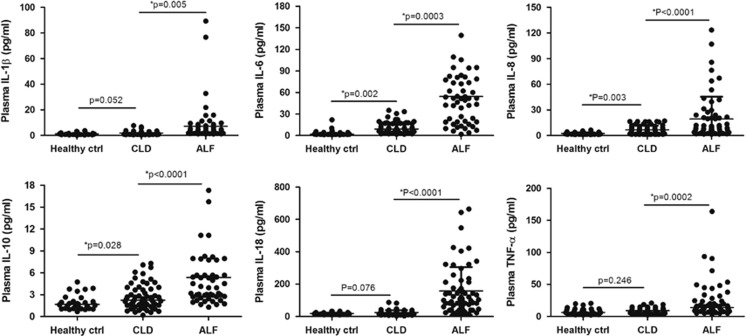
The occurrence of systemic inflammation in ALF patients at admission. Six cytokines (IL-1*β*, IL-6, IL-8, IL-10, IL-18, and TNF-*α*) representing a common range of pro-inflammatory and anti-inflammatory cytokines were measured in the plasma of ALF patients at admission. It showed that all six cytokines were significantly increased in ALF patients as compared with CLD patients or healthy controls. These cytokines in CLD patients showed a trend toward higher levels than in healthy controls, but only IL-6, IL-8, and IL-10 levels attained a statistical difference (*P<0.05). Variables were expressed as median (interquartile range)

There has been considerable evidence showing that the severity of systemic inflammation correlates with the severity of organ dysfunction and mortality rate, although we observed weak association between circulating histones and SIRS scores in ALF patients (Supplementary Table 1). We further assessed the degree of systemic inflammation in ALF patients at admission by measuring six cytokines (IL-1*β*, IL-6, IL-8, IL-10, IL-18, and TNF-*α*) in the circulation, which represent a common range of pro-inflammatory and anti-inflammatory cytokines. We found that all six cytokines were significantly increased in ALF patients as compared with the pathological controls or normal controls (Figure 2). These cytokines in CLD patients showed a trend toward higher levels than in the healthy controls, but only IL-6, IL-8, and IL-10 levels attained a statistical difference (all *P*<0.05). We made a correlation analysis of admission histone levels and these cytokines in ALF patients, and observed that plasma histone levels were significantly associated with IL-6 (*r*=0.692, *P*=0.005), IL-8 (*r*=0.537, *P*=0.031), IL-18 (*r*=0.618, *P*=0.012), and TNF-*α* (*r*=0.483, *P*=0.032), all of which are, indeed, important markers of systemic inflammation (Supplementary Table 2). Collectively, these results suggested that circulating histones were closely related to systemic inflammation during the onset of ALF, which might adversely affect disease progression such as multiple organ dysfunction and mortality.

### Circulating histones-mediated cellular damage and induced cytokine production

**Figure 3:**
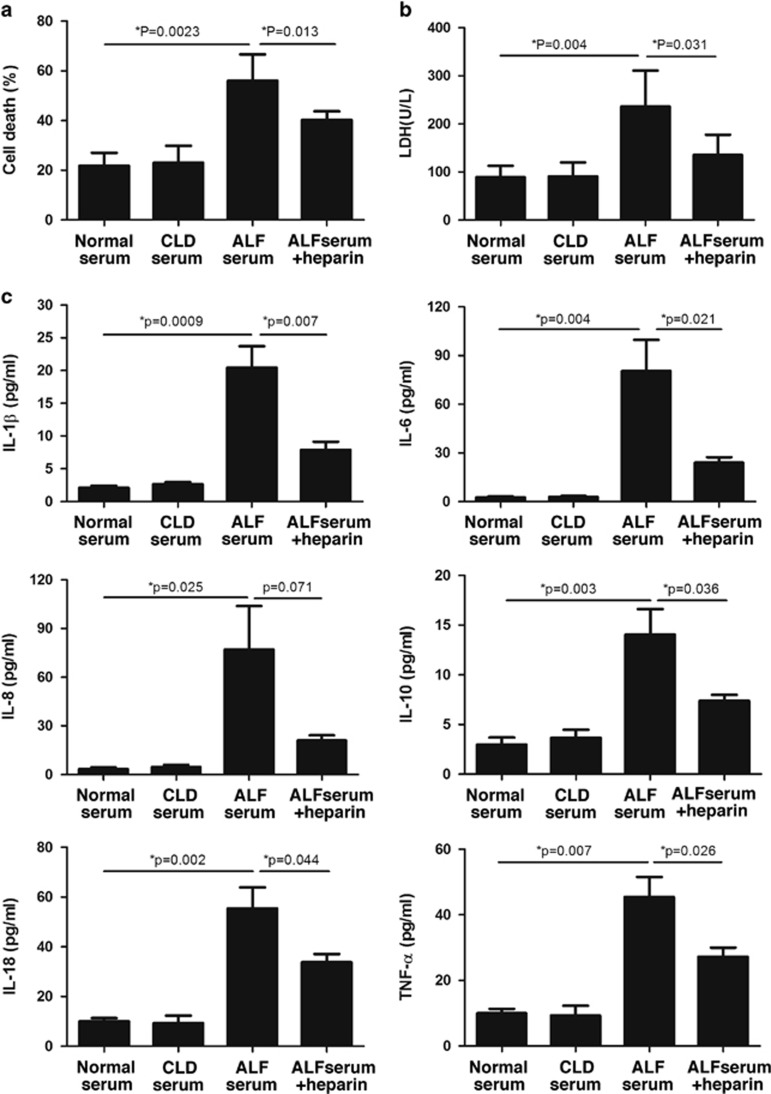
ALF patients' sera induced human L02 hepatocyte death and stimulated human monocyte U937 to produce cytokines. (**a**) It showed that ALF patients' sera could drastically decrease cell viability of human L02 hepatocytes after 24-h incubation as compared with CLD patients or healthy controls' sera, whereas addition of noncoagulant heparin could inhibit cell death caused by ALF patients' sera. (**b**) Lactate dehydrogenase (LDH) levels were significantly increased in supernatant of human L02 cells after stimulation of ALF patients' sera in contrast to the sera from CLD patients or healthy controls, whereas addition of noncoagulant heparin could reduce LDH levels. (**c**) The six cytokines were all notably increased in the supernatant of ALF serum-treated human monocytic U937 cells, whereas addition of noncoagulant heparin could decrease the levels of these cytokines. (*P<0.05) Variables were expressed as mean±S.D. The experiments were repeated at least three times

To further explore the pathogenic role of histones in the circulation of patients, we incubated ALF patients' sera with human L02 hepatocytes and human monocytes U937 cells, respectively. We observed that ALF patients' sera containing high concentrations of histones were obviously injurious to L02 cells, as evidenced by decreased cell viability and increased LDH levels (Figures 3a and b). Moreover, the stimulation of human U937 monocytes with ALF patients' sera led to a remarkable increase in histone-related cytokines (IL-1*β*, IL-6, IL-8, IL-10, IL-18, and TNF-α) in the supernatants of cell culture (Figure 3c). By contrast, the sera from CLD patients or healthy controls had little effects on both cells. The toxicity of the sera from ALF patients was not related to etiologies (data not shown), but mainly dependent on histone levels. To validate this, we added purified histones into normal sera and then administered to both cells. We observed that exogenous histones in normal sera achieved the similar cytotoxicity to both cells in a dose-dependent manner, which, thus, confirmed a pathogenic role of circulating histones (Supplementary Figures 1 and 2).

It was previously observed that targeting histones by specific neutralizing antibody (e.g., antihistone H4 antibody) or activated protein C (APC) was protective in several inflammatory conditions. In this study, we investigated whether noncoagulant heparin, which can bind histones, had some protective effects against histone-mediated cytotoxicity. We showed that the administration of noncoagulant heparin markedly inhibited ALF patients' sera-induced cell death or activation of U937 cells (Figure 3), which was demonstrated by improved cell viability and decreased inflammatory cytokine levels. This finding, thus, suggests a possible novel strategy for clinical intervention of ALF.

### Parallel elevation of circulating histones and inflammatory cytokines in mice with acute liver injury

**Figure 4:**
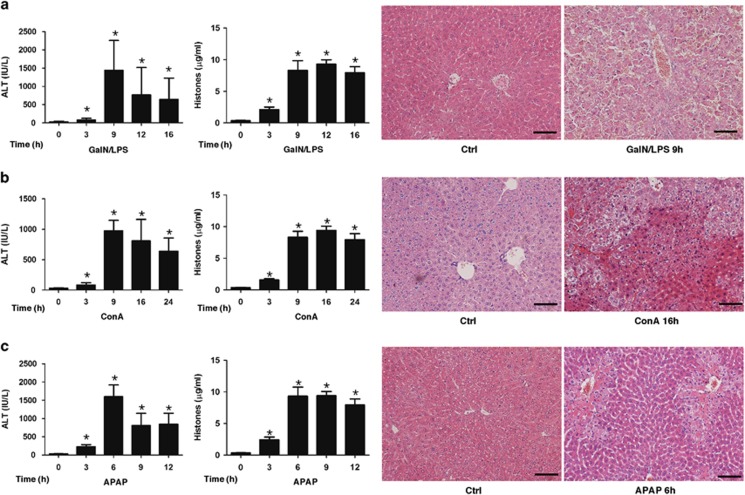
Elevation of circulating histones in mice with acute liver injury. (**a**) Administration of GalN/LPS to mice caused evident liver injury and a significant release of circulating histones. Plasma ALT levels were notably increased in a time-dependent manner following GalN/LPS injection to mice. Concomitantly, plasma histones in GalN/LPS-treated mice were increased remarkably in a time-dependent manner. The pattern was directional with the changes of ALT levels. The representative of H&E stained sections of liver damage at 9 h after GalN/LPS administration. Scale bars: 100 *μ*m. (**b**) ConA treatment via tail vein caused severe liver injury and a significant release of circulating histones in mice. The representative of H&E stained sections of liver damage at 16 h after ConA administration. Scale bars: 100 *μ*m. (**c**) APAP treatment caused acute liver injury and circulating histone release in mice. The representative of H&E stained sections of liver damage caused by APAP at 6 h. Scale bars: 100 *μ*m

To further translate our clinical and *ex vivo* findings into an *in vivo* system, we investigated whether circulating histones have a similarly toxic role, and whether they can be specifically targeted in animal models of acute liver injury. We used three well-established liver injury models by administering GalN/LPS, ConA, and APAP to mice, respectively, which represents most common etiologies of liver damage and mimics different causes of human ALF.^15, 16, 17, 18^ In these *in vivo* studies, we showed that acute liver damage was prominent around 6–16 h in all three animal models, as evidenced by increased serum ALT levels and typical histological alterations, and high circulating histone levels could be detected from 3 h after initiation of liver damage and reached a peak value between 9–12 hours (Figure 4). Concomitantly, there occurred a remarkable systemic inflammation in these mice with acute liver damage, as evidenced by significantly increased inflammatory cytokines including IL-1*β*, IL-6, IL-8, IL-10, IL-18, and TNF-*α* (Supplementary Figure 3). Of note, plasma histone levels correlated well with the concentrations of these cytokines (Supplementary Table 3). These data suggested that circulating histones-mediated systemic inflammation occurred evidently during the course of acute liver damage despite different causes, which are in line with our clinical observations.

### Noncoagulant heparin attenuates hepatic damage and systemic inflammation in mice

**Figure 5:**
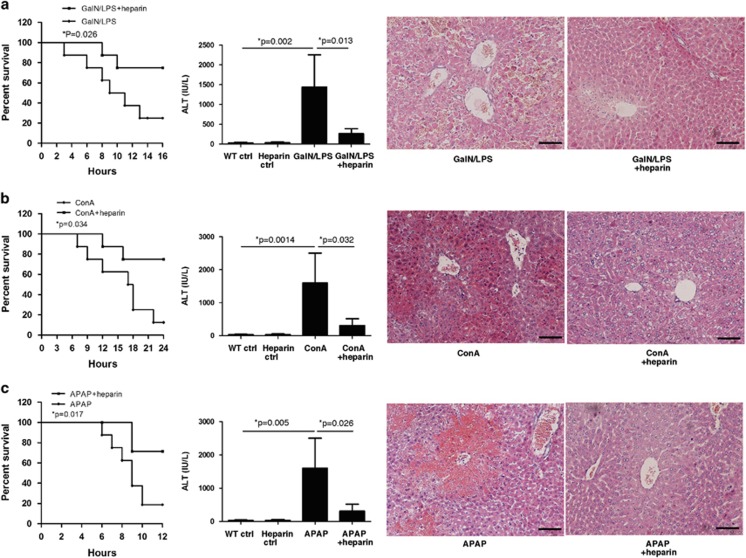
Noncoagulant heparin attenuates liver damage in mice. (**a**) Administration of noncoagulant heparin significantly reduced the mortalities of GalN/LPS-treated mice, and improved liver function. The representative of H&E stained sections of attenuated liver damage at 9 h after GalN/LPS plus heparin treatment. Scale bars: 100 *μ*m. (**b**) Administration of noncoagulant heparin significantly improved the survival rate of ConA-treated mice, and decreased the ALT levels. The representative of H&E stained sections of ameliorated liver damage at 16 h after ConA plus heparin treatment. Scale bars: 100 *μ*m. (**c**) Noncoagulant heparin significantly reduced the mortalities of APAP-treated mice, which was demonstrated by ameliorated liver function and histological alterations. The representative of H&E stained sections of attenuated liver damage at 6 h after APAP plus heparin treatment. Scale bars: 100 *μ*m

**Figure 6:**
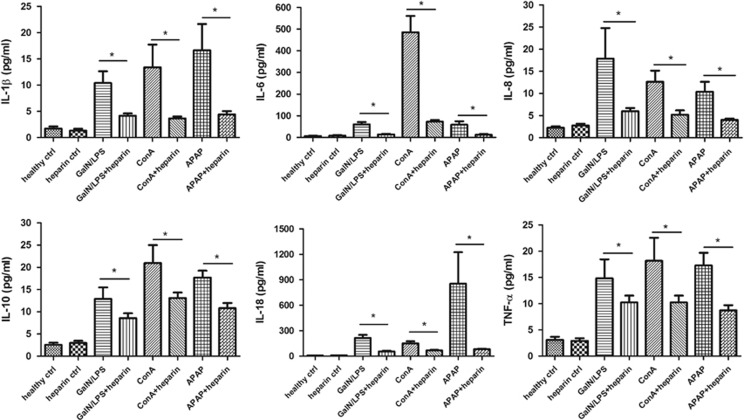
Noncoagulant heparin reduces systemic inflammation in mice. It showed that the administration of noncoagulant heparin significantly reduced cytokine production in all three murine models of liver damage (*P<0.05). Variables were expressed as mean±S.D.

As observed *in vitro* studies, noncoagulant heparin is able to inhibit histone-mediated cell death or stimulation of human monocytes. To determine whether the same is true for acute liver injury, we administered noncoagulant heparin to three models of mice by subcutaneous injection. We first compared the survival rate in heparin-treated or saline-treated mice with acute liver damage and then designated the measurement at predetermined time point to detect the protective effects of heparin. We observed that administration of noncoagulant heparin significantly reduced the mortalities of mice with acute liver injury of three models (Figure 5) and protected against liver damage despite different causes, which was demonstrated by ameliorated liver function and histological parameters, and significant reduction of cytokine release (Figure 6). We, thus, demonstrated that noncoagulant heparin could provide important protective effects on different etiologies-caused acute liver damage in mice, which mimic the feature of various causes of human ALF, through targeting circulating histones. These findings show a novel therapeutic potential for noncoagulant heparin in the treatment of ALF.

## Discussion

In this study, we described an important role played by circulating histones in the context of ALF in humans and mice. A key finding is that the presence of high-circulating histones correlates strongly with disease severity and mortality of ALF, thus indicating that circulating histones may serve as a novel biomarker with prognostic implications for ALF. In addition, significant association between high-circulating histones and high concentrations of pro-inflammatory cytokines, and SOFA, MELD scores suggests that the presence of systemic inflammation and concomitant organ dysfunction in ALF may be mainly attributed to large quantities of histones in the circulation of patients. To validate this speculation, we incubated ALF patients' sera with human hepatocytes or monocytes, and observed that histones in the circulation of ALF patients caused direct hepatocyte injury or stimulated innate cells, such as monocytes, to release cytokines. This finding was further consolidated by our demonstration of exogenous histones in normal sera achieving a similar cytotoxicity in a dose-dependent manner. Collectively, these clinically centered and *ex vivo* studies provide novel data, suggesting that massive release of histones into the circulation during the course of ALF may contribute significantly to cellular damage and systemic inflammation, which further promotes organ dysfunction and even death. Our data may uncover a novel view of the mechanisms related to ALF. Indeed, it has been reported that death of patients with ALF usually does not result from the primary hepatic insults but rather from subsequent insult events.^2^ Among these events, systemic inflammation seems to have a pivotal role in the outcome of ALF.^5, 19^ However, the causes or triggers of systemic inflammation in ALF are poorly defined and remain controversial. To the best of our knowledge, we for the first time identify circulating histones as a major culprit of host inflammation and organ dysfunction in ALF.

**Figure 7:**
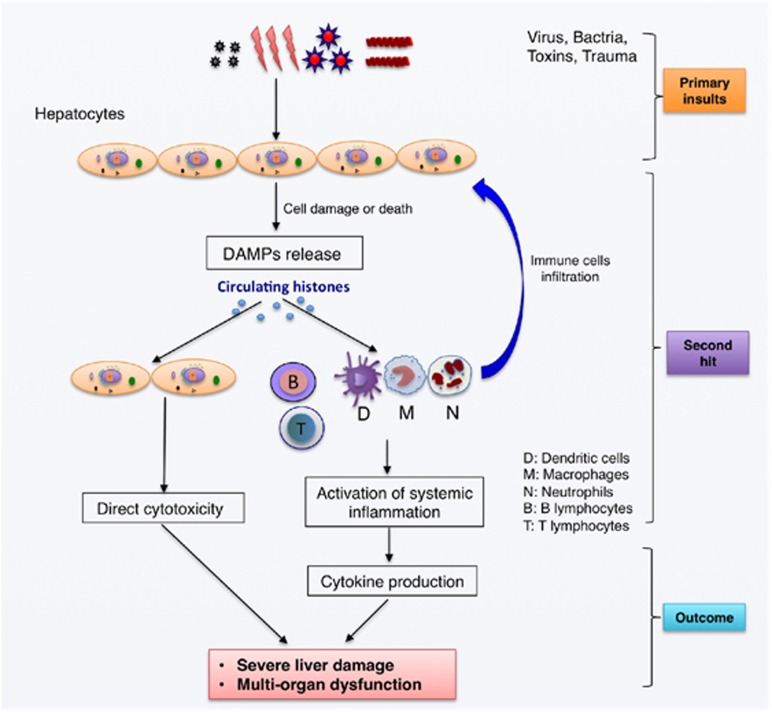
The hypothesized model of circulating histones in mediating systemic inflammation and liver injury. It is proposed that circulating histones may originate from cell damage or death caused by virus, bacteria, hepatotoxins, or trauma, then inducing direct cytotoxicity and activation of innate immunity and systemic inflammation, which in turn attracts more inflammatory cells and amplify inflammation by promoting cytokine production that eventually contribute to the pathogenesis of ALF

Histones are a group of nuclear proteins that form heterooctamers to wind up the double-stranded DNA to form a nucleosome.^11^ In some cases, elevated histone levels have been observed in the circulation of patients with myocardial infarction, stroke, infections, trauma, cancer, and autoimmune diseases.^20, 21, 22, 23^ However, whether circulating histones merely act as bystanders or are active mediators of the disease is uncertain. A recent study by Xu *et al.*^8^ showed that circulating histones are key mediators of cell damage and organ dysfunction during the hyperinflammatory reactions such as sepsis. Huang *et al.*^24^ studied the role of circulating histones in sterile inflammatory liver injury and showed that endogenous histones serve as a crucial link between initial tissue damage and activation of inflammation. Bosmann *et al.*^25^ demonstrated that circulating histones are essential effectors of C5aR- and C5L2-mediated tissue damage and inflammation in acute lung injury in humans. These studies indicate an importantly pathological and targetable role of circulating histones in systemic inflammation and organ dysfunction. In agreement with these findings, our data provided evidence that circulating histones may represent a novel pathologic mechanism for the initiation and progression of ALF. As to the source of circulating histones, we have previously demonstrated that this is most likely derived from massive destruction of liver cells and/or neutrophil extracellular traps.^26^ Moreover, we showed that the release of histones is not related to different causes of ALF, suggesting that histone-mediated cellular injury and inflammation are a common pathway implicated in the progression of ALF. We, therefore, propose a hypothetical model of circulating histones in mediating cellular injury and systemic inflammation in ALF (Figure 7).

Notably, the ability of histones to injure or stimulate cells *in vitro* can be prevented by noncoagulant heparin,^14^ which may provide a promising approach for management of ALF in clinical settings. Heparin is a glycosaminoglycan well known for its anticoagulant properties. Heparin also possesses anti-inflammatory effects and has been successfully used for the treatment of inflammatory conditions such as ischemia–reperfusion injury.^27, 28, 29^ However, the mechanisms responsible for its anti-inflammatory effects are not well understood. Indeed, heparin can bind many cellular proteins including histones through electrostatic interactions of high affinity. This occurs because heparin is highly sulfated and has strong negative charges whereas histones are positively charged.^30^ However, no one attributed the binding of heparin to histones to its anti-inflammation property before histones were revealed to be inflammatory mediators. As targeting histones by a neutralizing antibody or APC has proven to be protective,^8^ we explored whether heparin can interfere with histone-mediated cytotoxicity or inflammation in this study. However, as ALF patients are already at high risk of hemorrhage, a concern that heparin treatment could exacerbate disease complexity should not be ignored. To address this concern, we investigated the effect of nonanticoagulant N-acetyl heparin, which excludes the anticoagulant property while retaining its anti-inflammation ability. Wildhagen *et al.*^14^ confirmed that N-acetyl heparin has a strong affinity for histones and they observed a direct binding interaction between nonanticoagulant heparin and histones by using surface plasmon resonance and confocal laser scanning fluorescence microscopy. They also demonstrated that nonanticoagulant heparin has therapeutic potential to treat sepsis and other hyperinflammatory conditions in which the release of histones is evident.^14^ Here, after first demonstration of an inhibitory effect of noncoagulant heparin against histone-mediated cytotoxicity or stimulatory effects *in vitro* studies, we extended on our studies by using mouse models of acute liver injury to further examine whether the potential clinical link between histones and liver abnormalities could be reproduced and the disease could be specifically targeted by noncoagulant heparin *in vivo*. We used three well-established acute liver injury mouse models, which mimic different etiologies of liver damage clinically. For example, administration of GalN and LPS can induce liver damage that closely resembles human viral hepatitis in its morphological and functional features.^31^ ConA induces a T-cell activation-dependent inflammatory reaction, and massive necrotic liver injury and inflammation can be caused by the infiltration of activated T cells.^32^ APAP induces liver injury and lethality in mice mimicking drug-induced liver injury in patients with APAP overdose.^16^ Despite different causes and different mechanisms for hepatotoxicity, we demonstrated that circulating histones were unanimously significantly increased in these models, which were in line with clinical observations. Furthermore, we observed that elevation of circulating histones was strongly associated with massive release of inflammatory cytokines in mice, which confirmed a previous report that the release of inflammatory mediators followed by primary hepatic insult exacerbates systemic inflammation and organ dysfunction, and thus contributing to the outcome of ALF. More importantly, we showed that noncoagulant heparin had obviously protective effects against liver injury in these three mouse models. Heparin-treated mice had a significantly improved survival rate and reduced host inflammation, as well as improved liver histology compared with the control mice receiving hepatoxic agents only. It seemed that the protective effects of nonanticoagulant heparin were not due to its anticoagulant property but was dependent on its ability to bind histones. We, thus, raise the possibility that targeting histones by noncoagulant heparin may serve as a therapeutic option in patients with ALF.

In conclusion, we show that circulating histones may serve as a novel biomarker indicating the severity of illness and mortality in ALF. Besides, circulating histones may have a pathological and targetable role in the initiation and progression of ALF, with the possible mechanisms summarized in Figure 7. Neutralization of histones by noncoagulant heparin may be a potentially therapeutic strategy in the management of ALF in clinical practice.

## Materials and Methods

### Subjects

This study was a prospective observational study and was approved by the Ethics Committee of Beijing Youan Hospital, Capital Medical University, Beijing, China, and was performed with the Helsinki Declaration. Written informed consent was obtained from all patients or their next of kin before study inclusion.

A total of 62 patients with ALF admitted to the Department of Liver Diseases, Beijing Youan Hospital between April 2012 and May 2015 were prospectively recruited. Furthermore, 60 patients with chronic liver disease (CLD) were included to serve as pathological controls, and 30 healthy volunteers as normal controls. ALF in this study was defined as coagulopathy (prothrombin activity <40% or INR>1.5) and any degree of HE in a patient without previous underlying liver disease and with an illness of <26 weeks duration.^1, 4^ CLD refers to disease of the liver that had lasted over a period of 6 months and includes various etiologies-caused inflammation, liver cirrhosis, and hepatocellular carcinoma.^33, 34, 35^

Patients were investigated within 24 h of admission and followed continuously during hospital stay. Mortality was defined as death occurring within 28 days after diagnosis. Upon admission, peripheral blood samples were collected in serum gel and sodium citrate containing plastic tubes from all patients. Blood was then centrifuged at 2000 × *g* for 20 min at 4 ^°^C, and the generated serum and plasma were stored as aliquots at −80 ^°^C until analysis. Baseline characteristics, demographic details, and routine biochemical parameters of each subject were recorded. In addition, SIRS and MELD scores were calculated throughout admission.

### Measurement of circulating histones

As a nucleosome comprises 147 base pairs (bp) of DNA wrapping around a core of double-represented histone proteins H2A, H2B, H3, and H4, assaying the concentrations of nucleosomes allows for the relative quantification of histones.^25, 26^ The ELISA for detection of histones in plasma was from Roche (Mannheim, Germany), which uses a capturing antibody against an epitope shared by all histones and a detecting antibody against DNA. Purified mixed calf thymus histones (Sigma-Aldrich, St. Louis, MO, USA) were used to generate standard curves.

### Measurement of plasma cytokines

Plasma samples were analyzed for IL-1*β*, IL-6, IL-8, IL-10, IL-18, and TNF-α levels using the ProcartaPlex Multiplex Immunoassay from affimetrix eBioscience (San Diego, CA, USA), according to the manufacturer's protocol.

### Cell culture and treatment

The human hepatocyte cell line L02 and human monocyte cell line (U937) were obtained from the cell bank of Peking Union Medical College Hospital, maintained in DMEM (Dulbecco's Modified Eagle's Medium, Sigma-Aldrich) supplemented with 10% fetal bovine serum (HyClone, Logan, UT, USA), 2 mm glutamine, and 100 U/ml penicillin/streptomycin (Sigma-Aldrich) in a 5% CO_2_ humidified atmosphere at 37 °C.

The L02 cells and U937 cells were grown to around 80% confluence and then exposed to ALF patients' sera for 24 h, respectively. Sera from patients with CLD or healthy controls served as controls. In another set of experiments, normal sera were supplemented with different concentrations of purified histones (10–70 *μ*g/ml, Sigma-Aldrich), and then administered to L02 cells and U937 cells, respectively. For interventional studies, noncoagulant heparin (200 U/ml, Sigma-Aldrich) was added to cultured cells. Experiments were performed at least in triplicates.

### Assay for cell damage and stimulatory effects

Cell viability for L02 cells was first determined using the Cell Counting Kit-8 (CCK-8, Sigma-Aldrich), according to the manufacturer's instructions. Briefly, after being treated with the sera, the cells were incubated with 10 ml of CCK-8 solution for 1 h. The absorbance was detected at 450 nm using a spectrophotometer. In addition, LDH is a cytoplasmic enzyme and its activity reflects the degree of cell damage.^7^ LDH activities in L02 cell culture supernatant were measured after cells were treated with the sera with a commercially available kit (Roche), according to manufacturer's instructions. Absorbance was read at 490 nm using a spectrophotometer.

For the determination of stimulatory effects, after U937 cells being treated with the sera, the cell culture supernatants were analyzed for IL-1*β*, IL-6, IL-8, IL-10, IL-18, and TNF-*α* levels using the ProcartaPlex Multiplex Immunoassay from affimetrix eBioscience.

### Mouse models of acute liver injury and animal experiments

All the animal studies were performed under the protocols approved by the Animal Research Committee of Capital Medical University, Beijing, China. Ten-week-old male C57BL/6 mice were obtained from Capital Medical University Animal Breeding Unit and were housed in a room maintained at a constant temperature (25±2°C) and humidity (50±10%), with free access to food and water and subjected to a 12 h light/dark cycle. To induce acute liver injury, mice were given GalN (500 mg/kg) plus LPS (10 *μ*g/kg) by intraperitoneal injection, ConA (20 mg/kg) via tail vein injection, or APAP (500 mg/kg) by intraperitoneal injection, respectively. Nonanticoagulant heparin can bind histones and is recently reported to interfere with histone-mediated tissue injury.^14^ Thus, we administered nonanticoagulant heparin (300 U/kg) to mice subcutaneously to examine if it can provide some protective effects against liver damage. Mice were killed at predetermined time point to collect blood samples and liver tissues for further analysis.

### Statistical analysis

For human data, values were presented as medians and interquartile ranges. For animal or cell data, values were expressed as mean±S.D. Data were analyzed using unpaired Student's *t*-test or Mann–Whitney *U-*test (for two groups), one-way analysis of variance followed by Tukey post-tests (for more than two groups). Correlations between variables were assessed using Spearman's rank correlation or Pearson correlation analysis. The log-rank (Mantel–Cox) test was applied to perform animal survival analysis. Results were considered statistically significant when *P*<0.05. All statistical analyses were calculated using GraphPad Prism v5 (GraphPad Software, Inc., San Diego, CA, USA).

## Figures and Tables

**Table 1:** Baseline demographical and clinical characteristics, and outcome in patients with ALF, patients with CLD and healthy controls at admission

**Characteristics**	**ALF (*n*=62)**	**CLD (*n*=60)**	**HC (*n*=30)**
Age (years)	58.2 (43.8–76)	52.7 (34.8–78)	35.8 (28.9–52.7)
Sex (male/female)	45/17	32/28	17/13
			
*Primary injury*			
	Viral causes (HBV=28, HBV+HDV=8, HEV=6)	Chronic hepatitis B (30)	NA
	Drug toxicity (12)	Chronic hepatitis C (20)	
	Autoimmune hepatitis (6)	Liver cirrhosis (10)	
	Indeterminate causes (2)		
SIRS score	3 (1–3)	–	NA
SOFA score	9 (5–12)	–	NA
MELD score	39 (24–49)	9(4–14)	NA
HE grade			NA
Grade I or II (*n*)	37	–	
Grade III or IV (*n*)	25	–	
28-day survivors (*n*)	28	–	NA
Serum ALT (IU/l)	1052 (238.7–3296.8)	89.2 (67.7–176.5)	19 (10–45)
Serum TBil (μmol/l)	165.7 (79.2–398.2)	28 (14–65.7)	13 (7–45)
Serum creatinine (mmol/l)	197 (65–370)	68 (53–89)	NA
INR	3.9 (2.3–6.5)	1.5 (0.98–2.12)	NA

Abbreviations: ALF, acute liver failure; CLD, chronic liver disease; HC, healthy controls; INR, international normalized ratio; MELD, model for end-stage liver disease; SIRS, systemic inflammatory response syndrome; SOFA, sequential organ failure assessment. Variables are expressed as median (interquartile range)

## Abbreviations

ALF: acute liver failure
APAP: acetaminophen
CLD: chronic liver disease
ConA: concanavalin A
GalN/LPS: D-galactosamine plus lipopolysaccharide
HE: hepatic encephalopathy
INR: international normalized ratio
MELD: model for end-stage liver disease
SIRS: systemic inflammatory response syndrome
SOFA: sequential organ failure response syndrome
