# Supplementary material for: Circulating histones are major mediators of systemic inflammation and cellular injury in patients with acute liver failure
Source: Cell Death Dis. 2016 Sep 29;7(9):e2391–. doi: 10.1038/cddis.2016.303 (PMC5059889; doi:10.1038/cddis.2016.303)
Supplement: Supplementary Information [file cddis2016303x1.docx]

**Supplementary data**

**Circulating histones are major mediators of systemic inflammation and cellular injury in patients with acute liver failure**

Zongmei Wen^1*^, Zhen Lei^2*^, Lu Yao^3^, Ping Jiang^1^,Tao Gu^4^, Feng Ren^5^, Yan Liu^5^, Chunyan Gou^5^, Xiuhui Li^5^, Tao Wen^2#^

1.Department of Anesthesiology, Shanghai Pulmonary Hospital, Tongji University School of Medicine, Shanghai 200433, P.R. China

2.Medical Research Center, Beijing Chao-Yang hospital, Capital Medical University, Beijing 100020, P.R. China

3.Department of Forth Cadre, General Hospital of Beijing Military Command of Chinese PLA, Beijing 100700, P.R. China

4. Department of Oncology, First Hospital of Qinhuangdao, Qinhuangdao 066000, Hebei Province, P.R. China

5. Beijing Youan Hospital, Capital Medical University, Beijing 100069, P.R. China

***Zongmei Wen and Zhen Lei contributed equally to this work.**

#Correspondence should be addressed to Tao Wen, Ph.D., Medical Research Center, Beijing Chao-Yang hospital, Capital Medical University, Beijing, 100020, China; Email: [wentao5281@163.com](mailto:wentao5281@163.com)

**Supplementary Figure 1.**

Human normal sera were supplemented with different concentrations of purified histones (10-70 μg/ml), and then administered to L02 cells and U937 cells, respectively. It showed that exogenous histones in normal sera induced L02 cell death in a dose-dependent manner, and stimulated U937 cells to release various cytokines in a dose-dependent manner (* p<0.05 vs. the controls). Variables were expressed as mean±standard deviation (SD). The experiments were repeated at least three times.

**Supplementary Figure 2. The protective effects of anti-histone H4 antibody or noncoagulant heparin in exogenous histone-treated hepatocytes.** **(A)** The purified histones (50 μg/ml) were infused into human normal sera and then administered to L02 cells. At the same time, antihistone antibody (20 μg/ml) or noncoagulant heparin (200 U/ml) was given to the cells, respectively. After 16 h incubation, the cells were harvested and assayed for cell viability. **(B)** After 16 h incubation, cell culture supernatants were collected and analyzed for lactate dehydrogenase (LDH) levels with a commercially available kit (Roche, Germany), according to manufacturer’s instructions. Variables were expressed as mean±standard deviation (SD). The experiments were repeated at least three times.

**Supplementary Figure 3. Parallel elevation of various cytokines in mice with acute liver damage.** Administration of **(A)**GalN/LPS (**B**)ConA (**C**)APAP all caused a significant elevation of 6 cytokines in the plasma of mice, as compared with the controls (^*^p<0.05). Variables were expressed as mean±standard deviation (SD).
