# Supplementary material for: Circulating histones are major mediators of systemic inflammation and cellular injury in patients with acute liver failure
Source: Cell Death Dis. 2016 Sep 29;7(9):e2391–. doi: 10.1038/cddis.2016.303 (PMC5059889; doi:10.1038/cddis.2016.303)
Supplement: Supplementary Table 1 [file cddis2016303x2.docx]

|  | Histones in ALF (n=62) | |
| --- | --- | --- |
| Admission parameters | r | p |
| SIRS score | 0.107 | 0.577 |
| SOFA score | 0.491 | *0.035 |
| MELD score | 0.378 | *0.048 |
| INR | 0.414 | *0.041 |
| ALT(IU/L) | 0.515 | *0.033 |
| TBil (μmol/L) | 0.211 | 0.459 |
| creatinine (mmol/L) | 0.091 | 0.620 |

**Supplementary Table 1. Correlation of plasma histones with various variables in ALF patients at admission**

Correlations between variables were analyzed using Spearman's rank correlation or Pearson correlation analysis.

*p<0.05 was considered to be statistically significant.
