# Supplementary material for: Circulating histones are major mediators of systemic inflammation and cellular injury in patients with acute liver failure
Source: Cell Death Dis. 2016 Sep 29;7(9):e2391–. doi: 10.1038/cddis.2016.303 (PMC5059889; doi:10.1038/cddis.2016.303)
Supplement: Supplementary Table 2 [file cddis2016303x3.docx]

|  | Histones in ALF (n=62) | |
| --- | --- | --- |
| Admission cytokines | r | p |
| IL-1β | 0.207 | 0.398 |
| IL-6 | 0.692 | *0.005 |
| IL-8 | 0.537 | *0.031 |
| IL-10 | 0.314 | 0.134 |
| IL-18 | 0.618 | *0.012 |
| TNF-α | 0.483 | *0.032 |

**Supplementary Table 2. Correlation of plasma histones with various cytokines**

**in ALF patients at admission**

Correlations between variables were analyzed using Pearson correlation analysis.

*p<0.05 was considered to be statistically significant.
