# Supplementary material for: Circulating histones are major mediators of systemic inflammation and cellular injury in patients with acute liver failure
Source: Cell Death Dis. 2016 Sep 29;7(9):e2391–. doi: 10.1038/cddis.2016.303 (PMC5059889; doi:10.1038/cddis.2016.303)
Supplement: Supplementary Table 3 [file cddis2016303x4.docx]

|  | Histones in GalN/LPS-treated mice | | Histones in ConA-treated mice | | Histones in APAP-treated mice | |
| --- | --- | --- | --- | --- | --- | --- |
| cytokines | r | p | r | p | r | p |
| IL-1β | 0.431 | 0.157 | 0.812 | *0.006 | 0.542 | *0.009 |
| IL-6 | 0.689 | *0.008 | 0.783 | *0.004 | 0.762 | *0.006 |
| IL-8 | 0.576 | *0.041 | 0.621 | *0.025 | 0.506 | 0.063 |
| IL-10 | 0.510 | *0.042 | 0.435 | 0.068 | 0.632 | *0.035 |
| IL-18 | 0.658 | *0.013 | 0.768 | *0.022 | 0.669 | *0.028 |
| TNF-α | 0.738 | *0.002 | 0.709 | *0.001 | 0.617 | *0.029 |

**Supplementary Table 3. Correlation of plasma histones with cytokines in mice with acute liver damage**

Correlations between variables were analyzed using Pearson correlation analysis.

*p<0.05 was considered to be statistically significant.
