# Supplementary figures and images for: Circulating histones are major mediators of systemic inflammation and cellular injury in patients with acute liver failure
Source: Cell Death Dis. 2016 Sep 29;7(9):e2391–. doi: 10.1038/cddis.2016.303 (PMC5059889; doi:10.1038/cddis.2016.303)

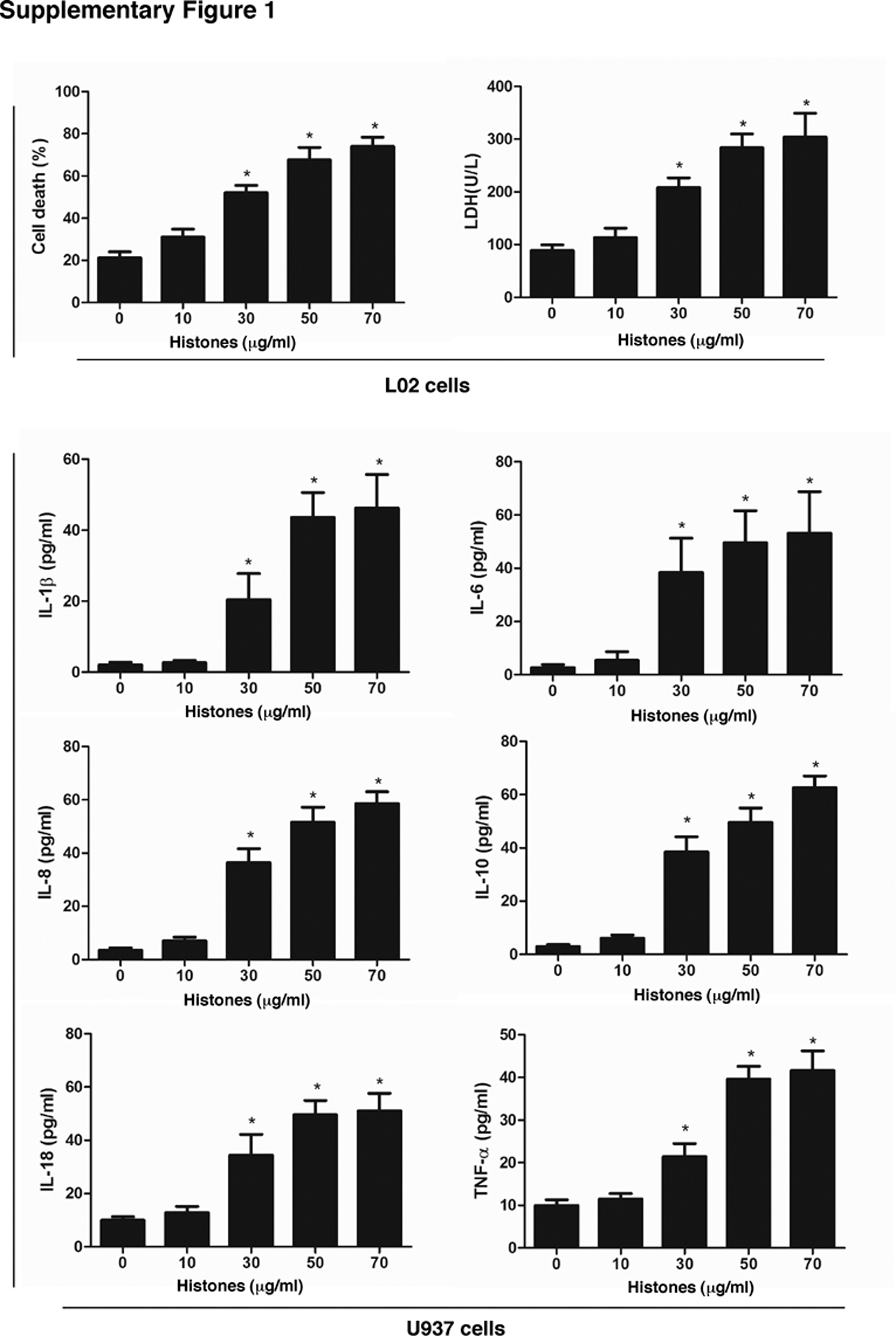

Supplement: Supplementary Figure 1 [file cddis2016303x5.tif]

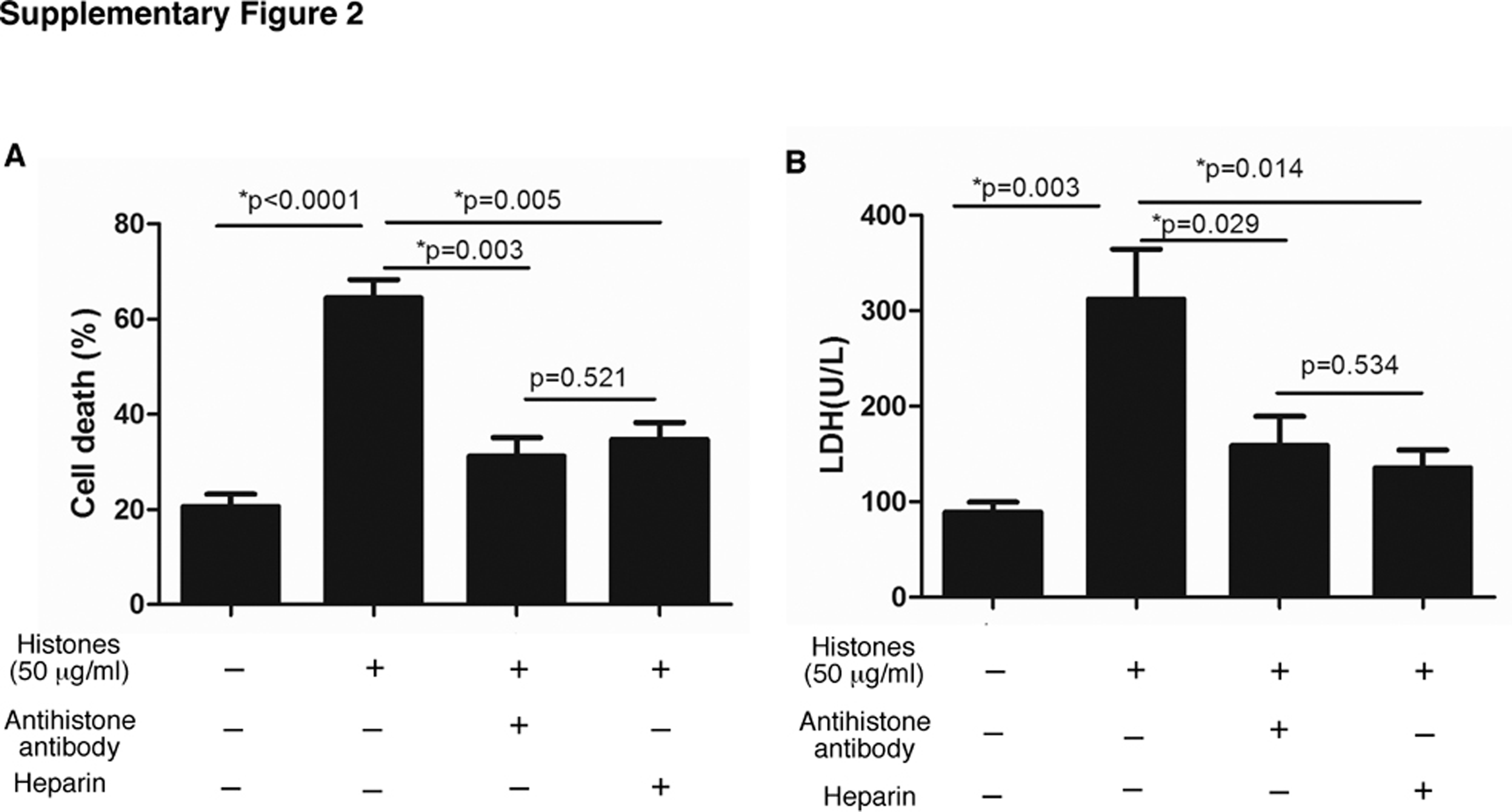

Supplement: Supplementary Figure 2 [file cddis2016303x6.tif]

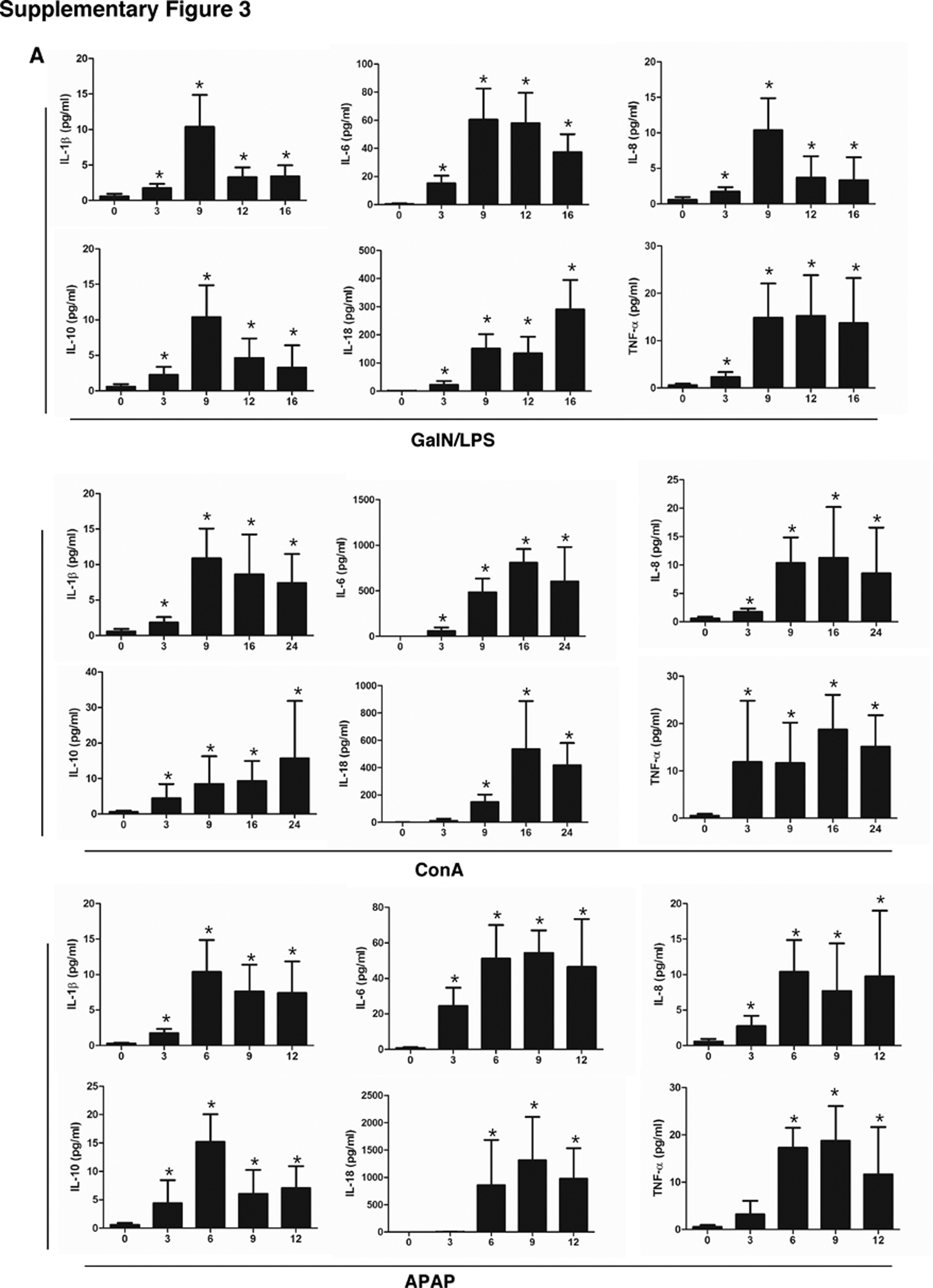

Supplement: Supplementary Figure 3 [file cddis2016303x7.tif]
